# Coagulopathy associated with COVID-19 – Perspectives & Preventive strategies using a biological response modifier Glucan

**DOI:** 10.1186/s12959-020-00239-6

**Published:** 2020-10-16

**Authors:** Nobunao Ikewaki, Kosagi-Sharaf Rao, Armando Durant Archibold, Masaru Iwasaki, Rajappa Senthilkumar, Senthilkumar Preethy, Shojiro Katoh, Samuel J. K. Abraham

**Affiliations:** 1 Department of Medical Life Science, Kyushu University of Health and Welfare, Nobeoka, Miyazaki Japan; 2 Institute of Immunology, Junsei Educational Institute, Nobeoka, Miyazaki Japan; 3 Instituto de Investigaciones Científicas y Servicios de Alta Tecnología (INDICASAT AIP), City of Knowledge, Panama City, Panama; 4 II Department of Surgery & Centre for Advancing Clinical Research (CACR), Yamanashi University- School of Medicine, Chuo, Japan; 5 The Fujio-Eiji Academic Terrain (FEAT), Nichi-In Centre for Regenerative Medicine (NCRM), Chennai, India; 6 Edogawa Evolutionary Laboratory of Science (EELS), Edogawa Hospital, Tokyo, Japan; 7 The Mary-Yoshio Translational Hexagon (MYTH), Nichi-In Centre for Regenerative Medicine (NCRM), Chennai, India; 8 GN Corporation Co. Ltd, Kofu, Japan

## Abstract

Direct endothelial injury by viruses and dysregulation of clotting mechanisms due to cytokine storm are the major precipitating factors of mortality in COVID-19; both are attributed to a fundamental dysregulation of the immune system. While immune dysregulation can be attributed to several factors, the risk of associated thrombogenic disruption varies across individuals. This variation depends on several factors, such as comorbidities, including diabetes, hypertension, and cardiovascular diseases. When considering ethnic variations, the vulnerability of Caucasians, African Americans and Hispanics needs to be addressed before arriving at strategies to handle thromboembolic complications, which have been identified in recent reports as the leading causes of mortality in COVID-19. Although evaluation of D-dimer and prothrombin during admission is considered to predict prognosis and mortality, there are no preventive or prophylactic strategies before hospital admission. Herein, we present our perspectives on the effect of regular supplementation with the biological response modifier beta glucan based on its relevance to immune modulation. This effect is of paramount importance in decreasing the development of severe COVID-19 and reducing mortality against the background of coagulopathy, especially in vulnerable populations.

## Introduction

Severe acute respiratory syndrome coronavirus 2 (SARS-CoV-2), the novel virus behind the coronavirus disease (COVID-19) pandemic, is wreaking havoc around the world [1]. Efforts are ongoing to understand the pathophysiological processes underlying the disease to mitigate complications. While severe acute respiratory distress syndrome is the major cause of death, other organ failures, such as acute kidney failure and acute cardiac injury, have also been associated with the disease [1].

The inflammatory response is highly increased during COVID-19 infection, and this process sets the stage for organ failure. Elevation of Th1 cytokine interferon (IFN)-gamma; inflammatory cytokines interleukin (IL)-1, IL-6, and IL-12; neutrophil chemokine IL-8; monocyte chemoattractant protein-1 (MCP-1); and Th1 chemokine IFN-gamma-inducible protein-1 [2] leads to a cytokine storm (CS) called macrophage activation syndrome (MAS) or secondary haemophagocytic lymphohistiocytosis (sHLH), which causes tissue damage [3]. Other immune dysregulation-related phenomena, including complement activation, also play a role in the organ failure caused by the virus. The host’s innate and adaptive immunity must come into play, encompassing different aspects, including the production of various pro-inflammatory cytokines and the activation of CD4 and CD8+ T cells for controlling viral infection and downregulating inflammation [3].

Coagulopathy has been reported in patients with SARS-CoV-2. Coagulation changes in patients with COVID-19 have been reported to include both disseminated intravascular coagulopathy (DIC) and sepsis-induced coagulopathy (SIC) [4]. During the early phase of COVID-19 infection, there is no clinical bleeding despite coagulation test abnormalities. Several factors, including certain treatments, have been found to culminate in the later development of SIC or DIC. The term COVID-19-associated coagulopathy (CAC) has been used to describe the coagulation changes occurring in COVID-19 patients [4]. Coagulopathy leading to venous thromboembolic events, end-organ failure secondary to a microangiopathy similar to disseminated intravascular coagulation (DIC), and stroke have all been reported in COVID-19 but with distinct features, wherein this coagulopathy in COVID-19 is associated with an increased risk of death [5]. However, it should be noted that even in the absence of advanced COVID-19, large artery stroke has been reported [6]. ACE2 receptors are expressed widely within endothelial cells, which could explain their vulnerability to SARS-CoV-2 binding, membrane fusion, and viral entry, thereby leading to infection and direct vascular injury [7] and giving rise to the coagulopathy-associated sequence in COVID-19.

Comorbidities such as diabetes, hypertension [8], and cardiovascular diseases [9] have been associated with a higher risk of complications and mortality due to COVID-19 [10]. Pre-existing increased plasmin activity occurring in hypertension, diabetes, and cardiovascular disease enhances the virulence and infectivity of the SARS-CoV-2 virus by cleaving its spike proteins, which in turn aggravates this coagulation-related process [11].

Herein, we report our perspectives on how people predisposed to coagulopathy and thrombogenic events could be the main target at high risk of complications due to COVID-19, and preventive measures can possibly be undertaken to allow predisposed people to successfully defend against complications due to COVID-19.

### Coagulopathy and COVID-19 – pathological mechanisms

In COVID-19, two separate pathologic processes have been found to play a role in producing clinical manifestations of coagulopathy: (i) local direct vascular and endothelial injury by invasion of the virus producing microvascular clot formation and angiopathy and (ii) mononuclear and polymorphonuclear infiltration along with apoptosis of endothelial and mononuclear cells as consequence of inflammation. Hypercoagulability with hyperfibrinogenaemia causing large vessel thrombosis and major thromboembolic sequelae should also be considered. The most common and critical feature observed in patients with coagulopathy-related predisposition to COVID-19 is abnormally elevated D-dimer levels. Elevated D-dimer levels have been associated with a poor prognosis. Increased prothrombin times (PTs) and activated partial thromboplastin times (aPTTs), lower platelet counts, and increased levels of lactate dehydrogenase (LDH) and ferritin are other factors associated with a poor prognosis in several studies [10].

A review article that provides guidance for the haemostasis laboratory in the management of the thrombotic risk associated with COVID-19 [12] suggests that the most seriously affected individuals have slightly longer PT than patients with more favourable prognosis. APTT is generally proportionally less prolonged than PT, probably due to an increase in plasma concentration of factor VIII and a low platelet count at admission (i.e., < 200 × 109/L), but it has also been associated with increased risk of death. The authors recommend monitoring the plasma concentration of PT, fibrinogen, D-dimers and platelet count every 48 h to continuously assess the thrombotic risk and identify timely warning signs of possible venous thrombotic events [12].

Hardy et al. [13] suggest that studies reporting on haemostasis in COVID-19 should ensure the careful reporting of laboratory methods to enable a correct interpretation of the thrombo-embolic risk and effects in COVID-19. For instance, a high variability in heparin plasma level measurement between the available measurement kits has been reported. The authors mention that the reagents designed to measure thrombin potentials require standardization. Some patients exhibit heparin resistance, which may be due to the normal thrombin generation profiles, which would have been reported as abnormal levels due to the methodologies employed. They also question whether viscoelastometric tests (VETs) recommended for studying thrombogenic profiles are truly standardized on a global scale [13].

Iba et al. [11] describe the four pathways of coagulation-related events and thrombus formation in COVID-19: (i) CS and pro-inflammatory cytokines such as interleukin (IL)-1β and IL-6 stimulate the expression of tissue factor on immune cells, thereby initiating extrinsic coagulation cascade activation; (ii) suppression of the fibrinolytic system by the decreased activity of urokinase-type plasminogen activator and increased release of plasminogen activator inhibitor-1; (iii) activation of platelets by various pro-inflammatory cytokines and the damaged endothelium readily binding with the activated platelets; and (iv) direct endothelial damage induced by inflammation.

### Coagulopathy and COVID-19 – incidence and vulnerable populations

Because the thrombogenesis associated with COVID-19 is complex and very little understood, a potential distinct “COVID-19-induced coagulopathy pattern” has been postulated [14]. The first report of haemostasis disorders in patients with COVID-19 was by Guan et al. on 28 February 2020 [15]. In this initial cohort of 1099 hospitalized patients with COVID-19, an increased D-dimer level above 0.5 mg/L was seen in 46.4% of the patients during initial presentation [15, 16]. In 191 hospitalized patients with COVID-19, 81% of non-survivors had D-dimer levels greater than 1 mg/L on admission [17]. A higher D-dimer level was identified in 59.6% of severe infections compared to 43.2% of non-severe COVID-19 patients. In fact, DIC has emerged as a strong predictor of mortality, with 71.4% of non-survivors meeting the criteria for DIC compared to only 0.6% of survivors [18]. Therefore, regular monitoring of D-dimer, prothrombin, and fibrinogen in COVID-19 is recommended because a significant increase in D-dimer and prothrombin with a decrease in fibrinogen has been observed at days 10–14 in non-survivors, and an elevated D-dimer level (above 1 mcg/mL) has been reported to be a strong independent risk factor in this vulnerable population [16, 19]. Caution should be exercised when interpreting plasma D-dimer values and unfavourable prognosis in COVID-19 because different thresholds have been proposed for stratifying the risk of mortality (i.e., between 1000 and 3000 ng/mL), and in the presence of significant pulmonary inflammation, fibrin deposits can occur within alveoli and the pulmonary extravascular space, whose lysis may also contribute to the rise of D-dimers [13]. Nevertheless, the working group on perioperative haemostasis considers patients with plasma D-dimer levels > 3000 ng/mL as having a very high thromboembolic risk; these patients might benefit from increased doses of heparin, and continued thromboprophylaxis is recommended after hospital discharge for a maximum of 45 days [20].

Other reports on the incidence of coagulopathy in COVID-19 include one with 150 patients with COVID-19, among whom 25 (16.7%) experienced a pulmonary embolism and two had three thrombotic circuit occlusions [21]. Lupus anticoagulants were detected in 50 of 57 patients tested (87.7%) [22]. Oxley et al. reported five patients with acute large vessel occlusion with ischaemic stroke [23]. In the original cases reported from Wuhan, China, stroke was seen in 5% of patients [24]. Another report indicated an incidence of thrombotic complications of 16–49% in patients with COVID-19 admitted to intensive care [13]. With regard to deep-vein thrombosis (DVT), of 143 patients hospitalized with COVID-19, 66 developed lower extremity DVT [25].

Because most of the COVID-19 coagulopathy data are from Chinese patients owing to the first reporting of COVID-19 being from China and because the incidence of venous thromboembolism is approximately 3- to 4-fold lower in Chinese patients [26], coagulopathy and thrombo-embolic events have been less important in Chinese hospitals, and the use of thromboprophylaxis is also less common. However, with the disease having affected Caucasian individuals at a magnitude several times greater than it has affected Chinese individuals, it is essential to know the ethnicity-related risk of thrombogenic events. Caucasians have higher thrombotic risk than Chinese and other Asian populations, and the risk is even higher in African-American and Hispanic patients in the USA [27–29]. Consistent with this pattern, a study conducted on COVID-19 coagulopathy in Caucasian patients showed that although the risk of coagulopathy was higher in Caucasians, because the patients included in the study were on low-molecular-weight heparin (LMWH) thromboprophylaxis, they rarely developed overt DIC, and in cases where DIC developed, it was during the later stages of the disease only. The study also reported a novel pulmonary-specific vasculopathy, which we term pulmonary intravascular coagulopathy (PIC), associated with COVID-19, that is distinct from DIC [30, 31]. Table 1 presents the race-based risk for COVID-19 coagulopathy, which confirms that the risk of coagulopathy is higher in Caucasians, African-Americans and Hispanics who should be treated with caution when affected with COVID-19 [32–40].

**Table 1:** Factors pre-disposing a specific Race/Ethnicity for increased risk of coagulopathy

S.No	Race/Ethnicity	Factor V Leiden andprothrombin gene polymorphism [32, 33]	Non-O Blood Group [32, 34]	Lower Protein C levels [32, 35, 36]	High levels of Procoagulant proteins FVIII andvon Willebrand factor [32, 37]	Elevated levels of D-Dimer [32, 38]	Undergoing surgery [32, 39]	Obesity [32, 40]
1	Caucasians/Europeans	High prevalence	High prevalence	High prevalence		High prevalence		
2	Afro-Americans	Intermediate prevalence			High prevalence		High prevalence	High prevalence
3	Hispanics					Intermediate prevalence		High prevalence
4	Africans							
5	Asians	Low prevalence		High prevalence (Japanese, Taiwanese and Thai)			Low prevalence	

Activation of innate immunity with older age and age-related coagulation cascade changes are also factors that have been reported to contribute to the vulnerability of elderly people to COVID-19 coagulopathy [41]. Alveolar macrophages (AMs) increase during ageing, but their plasticity to convert between pro- and anti-inflammatory states is greatly reduced. This accelerates COVID-19 in its early stages in the elderly and in advanced stages, causing excessive lung damage. A decline in neutrophil activity during ageing causes these cells to lose their ability to migrate to sites of infection and kill infected cells. The production and diversity of mucins and protective glycoproteins contributing to mucosal barriers also change during ageing [42]. The immunosenescence of the adaptive immune system in the aged also contributes to progression to severe COVID-19 in this population. A decline in the production of fresh naïve T cells, a less expansive T cell receptor (TCR) repertoire, T cell metabolic dysfunction, and weaker activation of T cells also contribute to immune vulnerability of the aged to COVID-19. An exploration of the link between the immune system and coagulopathy in the aged, which makes them a vulnerable population, identified that one in two fatal cases of COVID-19 experience a CS, among whom 82% are over the age of 60 [43]. Inflammaging is a major driver for this increased CS occurring in aged individuals, exacerbated by obesity, poor diet and oral health, microbial dysbiosis, and sedentary lifestyle [43]. Age-related correlation of higher basal circulating levels of pro-inflammatory cytokines, including IL-6, TNF-α, IL-1α, and CRP, is a reported phenomenon. During ageing, there is a gradual decline in immune function called immunosenescence and a chronic increase in systemic overactive but ineffective inflammation, a process known as inflammaging. Immunosenescence is characterized by impairment of both arms of immunity, innate and adaptive, and is characterized by ineffective pathogen recognition, macrophage activation, a reduction in natural killer (NK) cell cytotoxicity, thymic atrophy and accumulation of anergic memory lymphocytes [41]. The inflammaging process in older patients underlies the rapid progression to cytokine storms, whose key player is NLRP3, which is the major protein component of the inflammasome and is abundant in older individuals. NLRP3 priming induced by TLRs or tumour necrosis factor receptor activation leads to pro-inflammatory cytokine production, and activation is triggered by a range of stimuli that emerge during infections, such as tissue damage, nucleic acids, and invading pathogen proteins [41]. This process is further aggravated by decreased activity of sirtuin 2 (SIRT2) in the aged, as SIRT2 directly controls NLRP3 [41]. This cytokine storm disrupts the feedback control mechanisms of thrombin generation by antithrombin III, a tissue factor pathway inhibitor, and the protein C system, which predisposes individuals to the development of microthrombosis and DIC [43].

Coagulopathy seems to be the central factor predicting the progression of COVID-19. It also explains why children rarely experience severe illness due to COVID-19, as thrombotic complications in the paediatric age group are rare in the absence of an underlying cancer or a central venous access device. While pregnant women should be expected to have high vulnerability to coagulopathy, they have, in fact, been found to have only milder illness because of immune suppression during the gestational period to avoid foetal rejection, and thus, immunothrombosis does not come into play [43].

The vulnerability of patients with cardiovascular risk factors such as obesity, hypertension, and diabetes to the disease severity of COVID-19 is well established [7–9, 44]. Imbalance between coagulation and fibrinolysis with increased levels of clotting factors and relative inhibition of the fibrinolytic system, endothelial dysfunction, and enhanced platelet aggregation and activation, which are complications of diabetes, favour the development of a hypercoagulable pro-thrombotic state, thus explaining the high risk of diabetes patients to COVID-19 in terms of disease severity [44]. With regard to hypertension, other cardiovascular diseases, in addition to alterations in vascular and thrombogenic factors, pulmonary and peripheral endothelial injury due to direct viral attack, and CS have been indicated as inducers of hypercoagulation in these patients [45]. Another factor of concern is microparticles (MPs) [14]. These MPs are shed due to cell blebbing induced by activation of circulating blood cells, including platelets and leukocytes, and of endothelial cells by the COVID-19-induced cytokine storm. The membrane proteins on these MPs carry proteins and miRNAs to transmit the activator signal to distant cells propagating the disease and promote pro-coagulant responses due to the exposure of tissue factors and the activation of the coagulation cascade, ultimately leading to thrombin generation. Elevated levels of procoagulant MPs have been described in conditions such as arterial hypertension, diabetes mellitus, dyslipidaemia, obesity, pulmonary embolism, arterial hypertension, acute coronary syndromes, heart failure, etc. Thus, these individuals are at high risk of COVID-19-associated coagulopathy [14]. In the case of cardiovascular comorbidities, it has been hypothesized that damage mediated by the release of inflammation mediators acts on the vascular endothelium, leading to hypercoagulability and hypoxic damage by alterations of perfusion (acute coronary syndrome, thromboembolism, DIC) [46]. The immune response is also responsible for myocarditis with myocardial damage. Imbalance of the normal circulatory and inflammatory homeostasis due to alterations in the ACE2 receptor adds to the pathogenetic mechanisms of the damage, which is exacerbated by certain drugs used in people with hypertension, diabetes, and vascular disease, thus making these individuals at high risk of cardiovascular and thrombogenic events [46].

### Preventive and therapeutic aspects of coagulopathy in COVID-19

Having identified the various populations vulnerable to coagulopathy-associated severity of COVID-19, we now turn towards possible therapeutic solutions and preventive strategies. Recent guidelines recommend thromboprophylaxis for all hospitalized COVID-19 patients or full therapeutic-intensity anticoagulation [47]. Antiplatelet drugs, thrombolysis, immunomodulatory agents, and anti-complement drugs are suggested approaches. For anticoagulation, the drug of choice is low molecular weight heparin, and in patients who might have severe renal impairment or extremely high risk of bleeding, unfractionated heparin is recommended [48]. Preventive aspects deal with treating the comorbidities and end with a maximum level of thromboprophylaxis, but all of these measures are suggested after a patient is hospitalized. We explored whether any other preventive strategy is available. Supplementation with the biological response modifier beta glucan (BRMG) could be a solution in the vulnerable population. Beta glucans are potent biological response modifiers.

The effects of BRMG in infections and septic shock have been reported in both in vivo animal models and clinical studies [49]. BRMGs such as the soluble beta 1,3 glucans have been found to decrease septic complications and improve survival by acting on cytokine production and regulating coagulation activation [50] in a rat model. Preventive supplementation by BRMG prior to infection with *S. aureus* prevented sepsis in a guinea pig model [49]. Macrophage activation by BRMG has been shown to significantly reduce septic morbidity and mortality in human patients [51, 52]. In a randomized phase I/II trial, BRMG supplementation as an immune prophylaxis helped reduce postoperative infectious complications in patients undergoing major surgery by stimulating leucocytosis and phagocytic activity [52]. BRMG recognizes and interacts with the innate immune system in humans to help combat infections [53]. Radiation exposure and/or diabetes-induced oxidative stress, which causes disturbances in the measured clotting parameters by enhancing platelet aggregation and increasing thrombin levels, were reversed by yeast BRMG [50].

A BRMG from a black yeast, *Aureobasidium pullulans* (AFO-202), has been reported to be a potent immune modulator acting through its receptor, Dectin-1, which cooperates with pattern recognition receptors (PRRs) and Toll-like receptors (TLRs) in the innate immune response [54]. This BRMG reduces the levels of IL-1β, IL-2, IL-4, IL-6, IL-12, TNF-α, IFN-γ, and sFasL while increasing IL8 and sFAS [34], thereby balancing an effective optimal defence against viral infection without hyperinflammation. Anti-viral defence activities of BRMG occur through increased IL8, which causes activation, migration, and chemotaxis of neutrophils to kill virus-infected cells; increased type-I IFN production, which helps kill virus-infected cells; increased IL-7 production, which leads to development and survival of mature T-cells to maintain homeostasis; activation of B-cells, which results in production of virus-specific antibodies (IgG, IgM and sIgA) for neutralization of virus toxicity; and increased NK cell activity and macrophage activity [7]. Prevention of hyperinflammation occurs by preventing the onset of apoptosis through increased sFAS production, regulation and suppression of CS through activation of Treg cells and decreased IL6, and prevention of chemoattraction of monocytes and macrophages, T cells, NK cells, and dendritic cells through a decrease in CXCL10 and CCL2 (monocyte chemotactic protein 1; MCP-1) [7]. AFO-202 BRMG was found to enhance NK activity and cellular immunity in *Leishmania amazonensis*-infected mice [55]. AFO-202 BRMG has been demonstrated to protect mice infected with a lethal titre of the A/Puerto Rico/8/34 (PR8; H1N1) strain of influenza virus, and the mechanism of action was found to be through inhibition of viral replication [56].

**Fig. 1:**
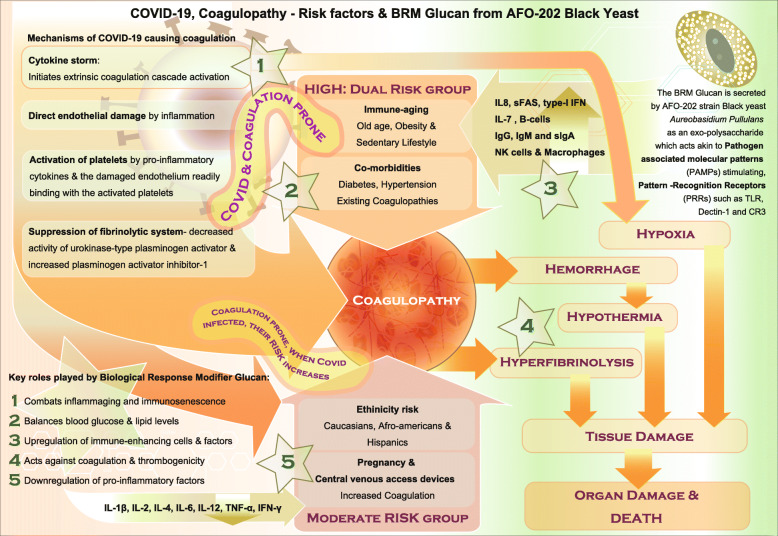
A schematic illustration of the implications of the COVID-19 coagulopathy cascade and the stepwise mechanism that leads to organ damage. Various risk factors modulated by the biological response modifier (BRM) glucan at various steps of this cascade are listed and graded

The effects of this BRMG in acting as a prophylactic supplement to help combat coagulopathy associated with COVID-19 are illustrated in Fig. 1.

SARS-CoV-2 infection triggers a cascade of events that result in a gradually increasing hyperinflammatory state, which in turn provokes thrombogenicity culminating in a potentially devastating microangiopathy, contributing to COVID-19-induced acute respiratory distress syndrome (ARDS) and organ damage [57]. AFO-202 BRMG has been proven to attenuate CS caused by pro-inflammatory cytokines such as IL-6 while mounting anti-viral defence [54] by upregulating NK cells, macrophages and factors such as IL8, sFAS, type I IFN, IL and antibodies. AFO-202 BRMG has also been proven efficacious in lifestyle diseases in East Asian and South Asian populations with maintenance of blood glucose and lipid levels [58–60]. Since comorbidities such as hypertension, diabetes mellitus, dyslipidaemia and obesity have a very strong correlation to coagulopathies due to the presence of a dysregulated inflammatory system [61], BRMG will be beneficial in decreasing the risk of coagulopathy in these individuals with dual risk (COVID-19-induced risk and comorbidity-induced risk). BRMGs have been reported to have direct antiplatelet, antioxidative, anticoagulant and antithrombotic actions, which further supports their beneficial effects as a supplement to prevent COVID-19-associated coagulopathy. Due to these attributes of BRMG, although it has not been tested in people with COVID-19 or those with ethnic or other predispositions to coagulopathy, it is worth considering its efficacy in controlling a pathophysiology that may trigger coagulopathy due to hyperinflammation.

## Conclusion

The proposed mechanisms for multiorgan dysfunction in COVID-19 are multifactorial, and a hypercoagulable state with micro- and macrocirculatory thrombosis has been identified as a key factor determining the clinical course and disease severity. Ethnically vulnerable populations such as Caucasians, African Americans, Hispanics, the elderly population, and patients with comorbidities are at high risk and require prevention during this hypercoagulable state. D-dimer and prothrombin have emerged as the most important biomarkers to be analysed at the time of hospital admission due to COVID-19. In the present scenario, where there is no definite pharmacological remedy for prevention or treatment presently available, a biological response modifier beta glucan food supplement that has several advantages in modulating the immune response is considered to be worth recommendation for clinical studies of COVID-19, especially in vulnerable populations.

## Data Availability

Not applicable.
